# Catalytic degradation of Orange II in aqueous solution using diatomite-supported bimetallic Fe/Ni nanoparticles†

**DOI:** 10.1039/c7ra13348k

**Published:** 2018-02-16

**Authors:** Naeim Ezzatahmadi, Teng Bao, Hongmei Liu, Graeme J. Millar, Godwin A. Ayoko, Jianxi Zhu, Runliang Zhu, Xiaoliang Liang, Hongping He, Yunfei Xi

**Affiliations:** Institute for Future Environments, Science and Engineering Faculty, Queensland University of Technology (QUT) Brisbane QLD 4001 Australia y.xi@qut.edu.au +61 731381995; School of Earth, Environmental and Biological Sciences, Science and Engineering Faculty, Queensland University of Technology (QUT) Brisbane QLD 4001 Australia; Laboratory for Nanominerals and Environmental Material, School of Resource and Environmental Engineering, Hefei University of Technology Hefei China; School of Chemistry, Physics and Mechanical Engineering, Science and Engineering Faculty, Queensland University of Technology (QUT) Brisbane QLD 4001 Australia; CAS Key Laboratory of Mineralogy and Metallogeny/Guangdong Provincial Key Laboratory of Mineral Physics and Materials, Guangzhou Institute of Geochemistry, Chinese Academy of Sciences Guangzhou 510640 China

## Abstract

A functional diatomite-supported Fe/Ni nanocomposite successfully remediated Orange II contaminant in aqueous solution. The hypothesis was that diatomite-supported Fe/Ni would not only be more effective than Fe/Ni but also require less metallic loading to effect the catalytic reaction. Batch experiments indicate that 99.00% of Orange II was removed using diatomite-supported Fe/Ni, while only 86.64 and 3.59% of Orange II were removed using bimetallic Fe/Ni nanoparticles and diatomite, after 6 h of reaction, respectively. Characterisation by X-ray diffraction (XRD), X-ray photoelectron spectroscopy (XPS), transmission electron microscopy (TEM) and scanning electron microscopy (SEM) indicates that the use of diatomite as a support material reduced the aggregation of bimetallic Fe/Ni nanoparticles, thereby resulting in an enhancement in the reactivity. A synergistic mechanism for the removal of Orange II by diatomite-supported Fe/Ni was proposed which involves adsorption, followed by catalytic reduction. This study has demonstrated that diatomite may be a suitable support material for stabilizing and dispersing bimetallic Fe/Ni nanoparticles and the resulting diatomite-supported Fe/Ni composite could be a promising catalyst for the remediation of dye-contaminated wastewater.

## Abstract 2

A functional diatomite-supported Fe/Ni nanocomposite successfully remediated Orange II contaminant in aqueous solution.

## Graphical abstract

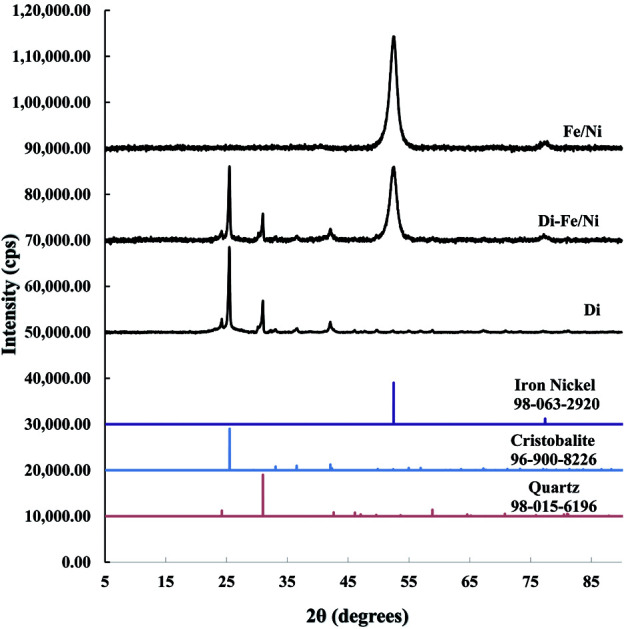

## 1 Introduction

Azo dyes are of major environmental concern owing to their toxicity, widespread usage, and bio-recalcitrance for conventional aerobic wastewater treatment.^1,2^ Azo dyes are complex organic molecules that are often difficult to degrade. They also may produce more hazardous intermediates during treatment processes. These latter reasons make the remediation of dye-contaminated water challenging. Of particular concern is the remediation of azo dye Orange II (Acid Orange 7). Orange II denoted as OII possesses a complex structure comprising two aromatic rings, an azo bond and a sulfonated group.^3^ It is mainly found in the wastewater of textile dying and cosmetic industries.^4^ OII is a poorly biodegradable azo dye, which is relatively stable and resistant to microbial attack.

For these latter reasons, various physical and biological processes have been developed for treatment of OII albeit there exist several limitations.^5–7^ For example, the effectiveness of physical processes, such as ion exchange or adsorption is not sufficient as they do not destroy pollutants, but transfer them from one phase to another.^8^ In biological processes, these dyes are not only non-biodegradable but also exhibit toxicity which inhibits biological treatment.^8^ To overcome the outlined disadvantages, advanced oxidation processes (AOPs) have been proposed.^3,9^ AOPs can facilitate degradation and in some cases complete mineralization of azo dyes in an efficient, fast and low-cost manner. Moreover, these processes have the advantage that no visible light or UV is required to initiate the reaction.^10^ Heterogeneous Fenton reaction using solid catalysts is a type of AOPs used for degradation of OII.^4,8,11^ Of particular interest is the use of nanoscale zero-valent iron (nZVI or Fe^0^).^12,13^ The use of nZVI in the Fenton reaction can significantly enhance the efficiency.^10^ Hydroxyl radicals (OH˙) generated through this reaction have a high standard oxidation potential (*E*^0^ = 2.73 V), which can oxidize many organic contaminants.^1,2^ However, this technology has inherent disadvantages, such as creation of a large amount of waste products (iron precipitate sludge) and requirement of low working pH (range of 2–3); which restricts its global acceptance as a practical wastewater treatment technology.^14,15^

Iron-based bimetallic nanoparticles have received attention in wastewater treatment technology^14,16,17^ owing to their small particle size, large effective surface area and inherent higher reactivity.^16^ The use of additional metallic catalysts, such as palladium (Pd) and nickel (Ni) with nZVI (Fe^0^) can enhance the rate of reduction by providing reactive electron donors or hydrogen catalysts. In bimetallic systems, nZVI plays the role of reducing agent and the second metal acts as a catalyst with hydrogen.^1,2^ These bimetallic nanoparticles offer advantages over nZVI, such as slower deposition of corrosion products and faster reaction rates.^1,2^ However, they have a strong tendency to aggregate into chain-like structures, significantly reducing their effective surface area and catalytic reactivity.^18^ In addition, bimetallic powder may lose its reactivity as it can be oxidised rapidly.

Silicate minerals, such as clay minerals and diatomite are candidates to act as support materials for bimetallic particles. Clays have proven to be suitable support materials for nZVI and bimetallic particles.^18–20^ Diatomite is another kind of amorphous silicate mineral which is comprised of fossilised skeletons of diatoms.^21^ It has received attention in wastewater treatment processes because of its attractive properties, such as high porosity and permeability as well as chemical inertness.^21^ It can be used as a support material for stabilizing and dispersing nZVI particles due to its highly porous structure, good mechanical and thermal stabilities.^21–23^ The porous structure also exhibits relatively high hydraulic conductivity which transfers contaminants to reactive sites, thereby increasing removal efficiency.^21^

To date, only a few studies have reported the use of clay-supported bimetallic nanocomposites for the remediation of contaminated aqueous solutions,^20,24–28^ while, to the best of our knowledge, diatomite-supported bimetallic nanocomposite has not been reported for the catalytic removal of organic contaminants. Therefore, the aim of this project is to create a novel diatomite-supported Fe/Ni (Di-Fe/Ni) composite material, which has high reactivity and efficiency for the removal of the azo dye Orange II (Acid Orange 7) as a model of organic contaminant. Hence, this study has focussed on the following research questions: (1) what is the structure of Di-Fe/Ni and Fe/Ni catalysts; (2) how does the catalyst composition impact OII removal; (3) what are the influences of pH, OII concentration, catalyst quantity, and reaction time on the removal of OII by Di-Fe/Ni and Fe/Ni catalysts; (4) what are the effects of different catalysts on the removal of total organic carbon (TOC); and (5) by what mechanism is OII removed from solution? To address the aforementioned questions, in this study, both bimetallic Fe/Ni nanoparticles and Di-Fe/Ni nanocomposites were synthesised and used to remove OII. A range of characterisation methods, including XRD, XPS, SEM, TEM, laser ablation inductively coupled plasma mass spectrometry (LA-ICP-MS) and micro-organic (CHNS) analysis were employed. A series of batch experiments were conducted to study the effects of different operational parameters toward removal of OII. Finally, a mechanism of OII removal by Di-Fe/Ni was discussed in this paper.

## 2 Materials and methods

### 2.1 Materials and chemicals

Diatomite is a courtesy of Mount Sylvia Diatomite Pty Ltd in Queensland, Australia. Orange II sodium salt, iron(iii) chloride hexahydrate (FeCl_3_·6H_2_O) and nickel(ii) sulfate hexahydrate (NiSO_4_·6H_2_O) were supplied by Sigma-Aldrich. Sodium borohydride (NaBH_4_) was purchased from Fluka Analytical. All chemicals are of analytical reagent grade and used without further purification.

### 2.2 Synthesis of Di-Fe/Ni and Fe/Ni

The raw diatomite was firstly purified by 1 M hydrochloric (HCl) acid solution. During this acid-leaching process, 20 g of diatomite was added into 1 L of HCl solution and stirred continuously overnight. Then, the solid product was separated through filtration and washed several times by deionised water until HCl solution was completely removed. The final product was denoted as “Di”.

Fe/Ni nanoparticles and Di-Fe/Ni nanocomposite were synthesised through liquid-phase reduction method, similar to previous studies.^16,26^ In brief, 9.65 g of FeCl_3_·6H_2_O and 0.90 g NiSO_4_·6H_2_O were dissolved in miscible liquids (37.5 mL ethanol plus 12.5 mL deionised water) and stirred for 20 min. Then, 2 g of Di was added to the aforementioned bimetallics mixture and stirred for 2 h under nitrogen atmosphere. Next, 4.16 g of NaBH_4_ was dissolved in 100 mL of deoxygenated deionised water to form 1.1 M NaBH_4_ solution. This solution was added dropwise to the mixture of Di and bimetallics and then stirred for 2 h in a fume hood. Fe^0^ and Ni^0^ particles were produced through the following reactions (eqn (1) and (2)).^26,29^

1 4Fe^3+^ + 3BH_4_^−^ + 9H_2_O → 4Fe^0^↓ + 3H_2_BO_3_^−^ + 12H^+^ + 6H_2_↑

2 Ni^2+^ + 2BH_4_^−^ + 6H_2_O → Ni^0^↓ + 2B(OH)_3_ + 7H_2_↑

Afterward, the solid product was separated from the solution and washed three times with 500 mL of absolute ethanol. The sediment was then dried at 60 °C. The resulting nanocomposite material was denoted as “Di-Fe/Ni” and kept in a vacuum desiccator before use. The unsupported Fe/Ni sample was synthesised through an identical procedure but without diatomite.

### 2.3 Characterisation

X-ray diffraction (XRD) patterns of Di, Fe/Ni and Di-Fe/Ni were obtained using a PANalytical X'Pert PRO 240 mm radius diffractometer with a Co-Kα radioactive source (*λ* = 0.179 nm) operating at 40 kV/40 mA. All samples were scanned with a fixed divergence slit of 0.5° from 5° to 90° (2*θ*). A Thermo Scientific spectrophotometer with Al K Alpha source gun type was used for X-ray photoelectron spectroscopy (XPS). High resolution analysis was carried out with pass energy of 30 eV at energy step size of 0.05 eV and an analysis area of 400 μm^2^.

Morphological studies involved use of a scanning electron microscope (SEM) (Zeiss Sigma, FESEM). All samples were coated with gold before analysis. Moreover, a JEM 2100 transmission electron microscope (TEM) operating at 200 kV was employed to analyse the microstructure of Fe/Ni and Di-Fe/Ni samples.

Elemental analysis was carried out by a LECO TruSpec Micro organic (CHNS) analyser. The samples were heated to 1050 °C in the presence of oxygen and the resulting gases were analysed using either infrared or thermal conductivity. This machine was calibrated with sulfamethazine and the analysis was conducted in triplicate. Moreover, the Laser Ablation Inductively Coupled Plasma Mass Spectrometry (LA-ICP-MS) technique was used to investigate the distribution of the elements in Di-Fe/Ni composite. In this study, an Agilent 8800 single collector, quadrupole attached to a 193 nm wavelength excimer laser with two-volume cell was employed. The LA-ICP-MS analysis was conducted in duplicate.

### 2.4 Batch experiments for the degradation of OII

OII degradation experiments were performed at its natural pH (∼6.84) and room temperature using Fe/Ni and Di-Fe/Ni. In a typical experiment (study of pH effect, catalyst amount and initial dye concentration on OII removal), 0.1 g of each sample was added to a 50 mL beaker containing 25 mL of 100 mg L^−1^ OII solution. Then, the mixture was stirred for 6 h. To undertake degradation kinetic studies, 1.0 g of sample was added into 250 mL of 100 mg L^−1^ OII solution. Mixed solution was left at its initial pH level stirred to the desired time intervals (10, 20, 30, 40, 50, 60, 120, 180, 240 and up to 600 min) in order to determine the minimum time to attain the equilibrium concentration. After the reaction, the pH of the solution was immediately recorded. Then, the solid product was separated using centrifugation at 3000 rpm for 20 min. Next, the supernatant was withdrawn using a syringe with a hydrophilic PTFE filter (0.22 μm). Several runs were performed by varying initial pH (3–11), catalyst amount (0.025–0.200 g), and initial OII concentration (50–200 mg L^−1^). All OII degradation experiments were conducted in duplicate.

The concentration of OII was measured spectrophotometrically using an Agilent Cary 60 UV-Vis Spectrophotometer by measuring absorbance at *k*_max_ of 485 nm. The removal efficiency of OII using materials was calculated by eqn (3):3
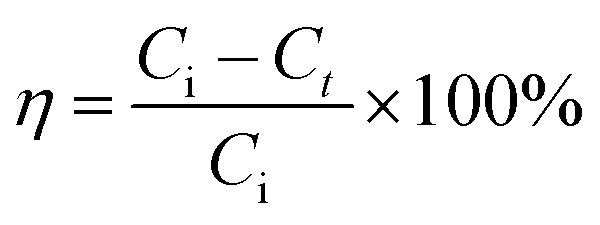
where *η* (%) = OII removal efficiency, *C*_i_ = initial OII concentration (mg L^−1^), *C*_*t*_ = OII concentration at *t* min (mg L^−1^).^24^

Moreover, the concentration of total organic carbon was measured by the use of a Shimadzu TOC-V total organic carbon analyser. The furnace was set to 720 °C and the carrier gas was zero grade air.

## 3 Results and discussion

### 3.1 Characterisation of Di, Fe/Ni and Di-Fe/Ni catalysts

#### 3.1.1 X-ray diffraction

**Fig. 1:**
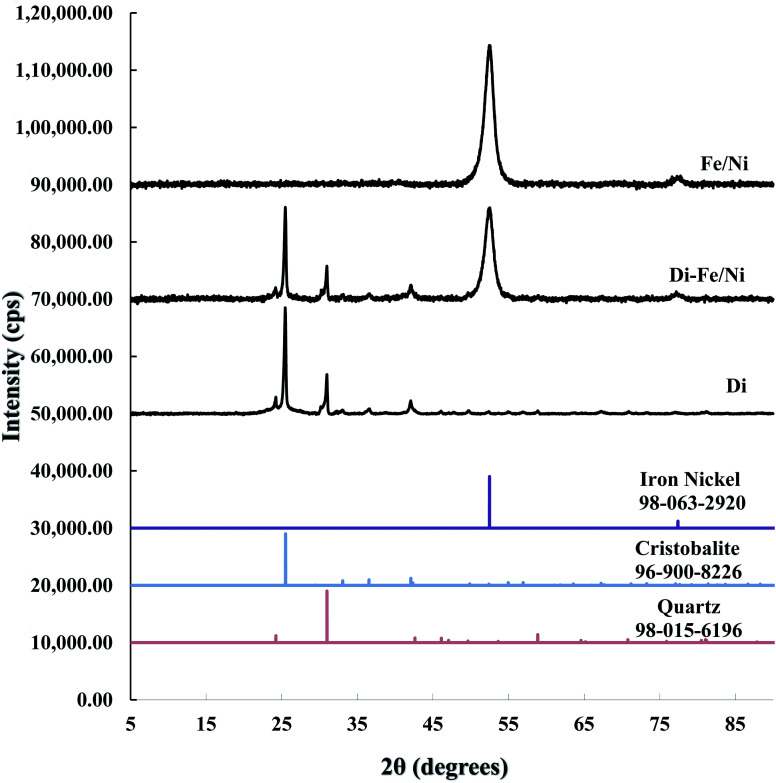
XRD patterns of Di, Di-Fe/Ni, and Fe/Ni.

XRD patterns of Di, Fe/Ni and Di-Fe/Ni are shown in Fig. 1. The XRD pattern of Di is similar to the pattern for amorphous Opal-A, which has a strong reflection (2*θ* ∼ 26°), and also displays minor reflections representing the inherent structure of diatomite.^30^ The XRD pattern of Di is not identical to data reported using a different type of diatomite.^21^ It is noteworthy that in previous literature,^30^ Cu-Kα radioactive source (*λ* = 0.154 nm) was used; while, in this study Co-Kα radioactive source (*λ* = 0.179 nm) was employed (to ensure better resolution). Hence, based on Bragg's law, *d* values for both analyses were calculated for comparison. As a result, the reflection with a *d* value at 0.403 nm (2*θ* ∼ 22°) using Cu-Kα source^30^ was comparable to the *d* value using Co-Kα source (*d* = 0.397 nm, 2*θ* ∼ 26°) used in this study, indicating the similarity in the results for both analyses.

A combination of cristobalite (96-900-8226) and quartz (98-015-6196) matches with the XRD patterns of Di and Di-Fe/Ni. Both patterns show two broad reflections at 2*θ* ∼ 26° and 31°, which are attributed to the cristobalite and quartz phases, respectively. The XRD pattern of Fe/Ni is in good agreement with a previously reported study.^18^ There are two reflections at 2*θ* ∼ 52° and 77° assigned to iron nickel (98-063-2920). The XRD pattern of Di-Fe/Ni also shows these reflections; where broad peak at 2*θ* ∼ 52° indicates that Fe^0^ and Ni^0^ nanoparticles are present in Di-Fe/Ni composites,^18^ and peak at 2*θ* ∼ 77° is relative to the presence of Fe_2_O_3_. Comparing the patterns of Di-Fe/Ni and Di, no considerable changes were observed before and after modification with Fe/Ni. This result may reflect the low level of loading and crystallinity of Fe/Ni nanoparticles.

#### 3.1.2 X-ray photoelectron spectroscopy

**Fig. 2:**
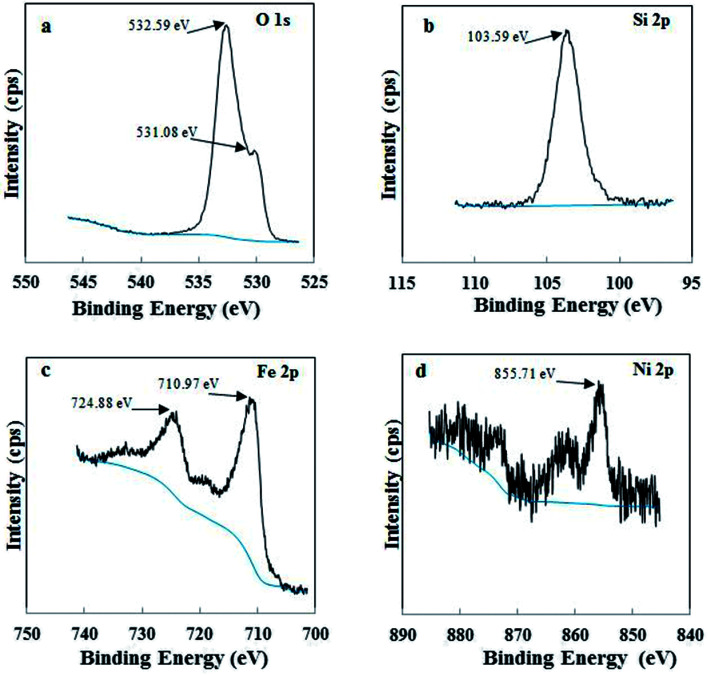
XPS spectra of Di-Fe/Ni composite: O 1s (a), Si 2p (b), Fe 2p (c) and Ni 2p (d).

XPS was used to examine the different chemical environments of the elements present in the catalyst. High resolution XPS spectra of O 1s, Si 2p, Fe 2p and Ni 2p for Di-Fe/Ni composite are displayed in Fig. 2. For XPS analysis, peak position was calibrated using C 1s spectra (Fig. S1†), which shows a strong peak at 284.80 eV. As shown in Fig. 2a, there are one weak peak at 531.08 eV and a strong peak at 532.59 eV, attributed to O 1s binding energy of SiO_2_ from diatomite structure.^21^Fig. 2b shows the photoelectron peaks of Si 2p and in this case only one strong peak at 103.59 eV was detected, which is ascribed to siloxane groups (Si–O–Si) of diatomite. These results are in line with those recently reported in the literature.^23^ In addition, there are two strong photoelectron peaks of Fe 2p at 710.97 eV and 724.88 eV (Fig. 2c), demonstrating the presence of a layer of iron oxide on the surface of Di-Fe/Ni.^16,31^ This data indicates that the surface of Di-Fe/Ni composite is covered by oxide layers formed during synthesis of the composite.^31^ On the other hand, there are some weak peaks (706.57 and 719.13) in this spectrum, suggesting the formation of Fe^0^. However, these peaks could not be easily observed due to oxidation of iron particles on Di-Fe/Ni. As seen from Fig. 2d, there is one broad peak at 855.71 eV in the XPS spectrum of Ni 2p_3/2_, which suggests the presence of Ni^0^ on Di-Fe/Ni.^31^

#### 3.1.3 Electron microscopy

**Fig. 3:**
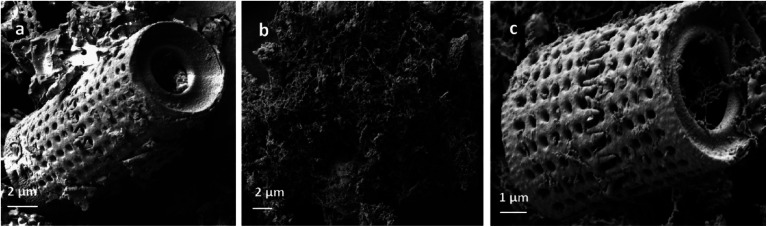
SEM images of Di (a), Fe/Ni (b) and Di-Fe/Ni (c).

SEM analysis was employed to investigate surface morphology and morphological differences in Di, Fe/Ni and Di-Fe/Ni (Fig. 3). The SEM image of Di is similar to those in previously reported studies which used the same diatomite source.^22,23^ However, it is noted that diatomite shape can differ if alternate types of diatomite are employed.^21,30^ Di frustules are mostly of cylindrical shape with diameters *ca.* 4 to 8 μm (Fig. S2†). As observed in Fig. 3a, a hollow cylinder of Di exhibits a well-developed porous structure. The pores in the cylinder are approximately 350 to 600 nm in diameter. Fig. 3b depicts the chain-like structure of bimetallic Fe/Ni nanoparticles, indicating that these nanoparticles have a strong tendency to aggregate. This aggregation may be caused by magnetic attraction between Fe and Ni nanoparticles. However, after grafting Fe/Ni on the surface of Di, the aggregation of these nanoparticles significantly decreases, as can be seen from the SEM image of Di-Fe/Ni (Fig. 3c). Fig. 3c also shows the dispersion of bimetallic Fe/Ni into the pores and on the surface of diatomite. Moreover, the porous structure of Di-Fe/Ni composite remains clearly visible, demonstrating the low loading amount of Fe/Ni, as indicated by XRD analysis. This was also confirmed by LA-ICP-MS analysis used to investigate distribution of the elements. As seen in Table 1, the mass percentages of Fe and Ni in the synthesized Di-Fe/Ni composite are 14.75 and 0.45%, respectively, confirming the low loading amount of the bimetallic particles in the composite.

**Table 1:** Element distribution on Di-Fe/Ni composite

Sample	Mass percentage (wt%)		
	1^st^ run	2^nd^ run	Average
Fe	14.79	14.71	14.75
Ni	0.47	0.43	0.45

**Fig. 4:**
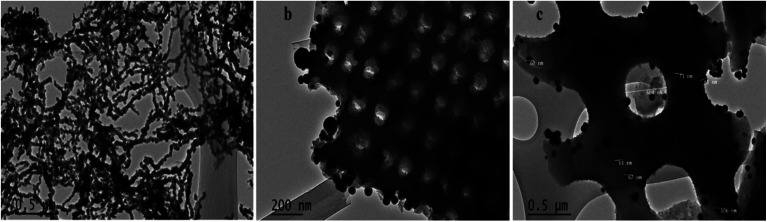
TEM images of Fe/Ni (a) and Di-Fe/Ni (b and c).

TEM was used in this study to expand the observations obtained from SEM by gathering more precise information about the surface of Di-Fe/Ni and Fe/Ni samples (Fig. 4). Fig. 4a displays chain-like structure of Fe/Ni nanoparticles, which indicates the aggregation of these particles, similar to that observed in Fig. 4b. However, when Fe/Ni nanoparticles were loaded on the surface and into the pores of Di, significant changes were observed (Fig. 4b). Fe/Ni nanoparticles were separated and displayed as individual spherical particles. In addition, Fig. 4c shows that the sizes of Fe/Ni nanoparticles in the pores and on the surface of Di are in the range 50–80 nm.

### 3.2 Degradation of OII using different catalysts

**Fig. 5:**
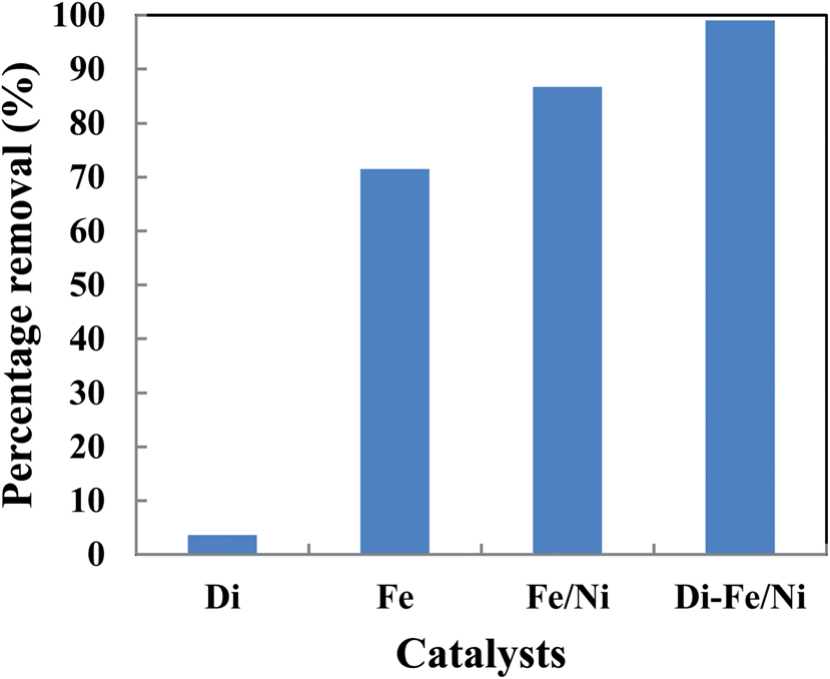
The degradation of OII in aqueous solution using different catalysts (initial concentration of OII: 100 mg L^−1^; volume of OII solution: 25 mL; catalyst amount: 0.1 g; pH: 6.84; reaction time: 6 h).

The removal of OII in aqueous solution using Di, Fe (nZVI), Fe/Ni and Di-Fe/Ni was investigated, with results shown in Fig. 5. 99.00% of OII was removed by Di-Fe/Ni nanocomposite, while 86.64% of OII was removed by bimetallic Fe/Ni nanoparticles. This result suggests that the aggregation of Fe/Ni was decreased (as demonstrated by TEM) by the use of Di as a support material; hence, the reactivity of Fe/Ni was increased due to the greater surface area of the active species. The removal of OII by Fe is 71.50%, which is lower than that of Fe/Ni. This behaviour may reflect the fact that use of additional metallic catalysts, such as Ni can improve the reactivity of Fe by providing reactive electron donors or hydrogen catalysts.^1,2^ The results also indicate that Di alone does not exhibit significant OII removal, as only 3.59% of OII was removed through presumably adsorption. This low adsorption capability may be due to the presence of negative surface charge of Di which repels negative sulfonate groups of OII. However, the removal of OII was significantly increased when Di was modified by Fe/Ni nanoparticles on the surface of Di-Fe/Ni.

### 3.3 Batch experiments

#### 3.3.1 Effect of pH on OII degradation

**Fig. 6:**
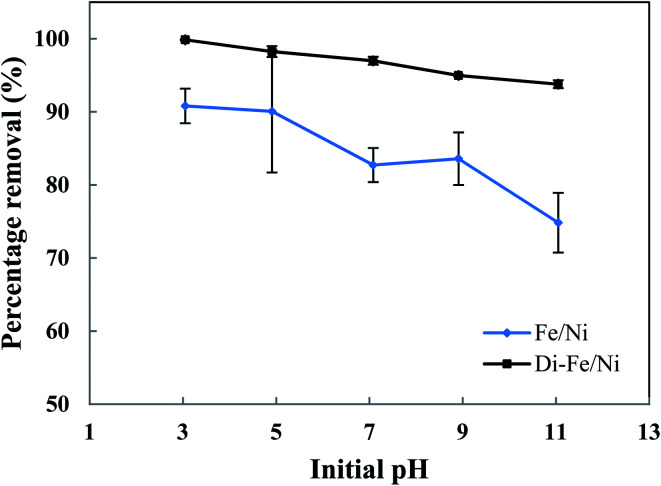
Effect of initial pH on OII removal using Fe/Ni and Di-Fe/Ni (initial concentration of OII: 100 mg L^−1^; volume of OII solution: 25 mL; catalyst amount: 0.1 g; reaction time: 6 h).

The influence of initial pH on removal of OII by Di-Fe/Ni and Fe/Ni catalysts was studied at ambient temperature by using 0.1 g of the catalyst with 25 mL of approximately 100 mg L^−1^ OII solution (Fig. 6). Initial pH values of the solution were varied from 3 to 11 using either 1 M NaOH or 1 M HCl. It is noteworthy to mention that the amount of OII adsorbed by the syringe filter and centrifuge tubes was found to be negligible. Moreover, as acid-leached diatomite (Di) adsorbed only a negligible amount of OII (3.59%), no further adsorption studies were performed using Di.

As seen in Fig. 6, pH of the solution has a significant impact on the degradation of OII. The OII removal efficiencies by Di-Fe/Ni are considerably higher than those for Fe/Ni, particularly at pH > 7. The results indicate that OII was completely removed by Di-Fe/Ni at pH 3, while 90.81% of OII was removed by Fe/Ni at the same pH value. However, for both samples, the OII removal efficiency increases with the decrease in the solution pH. Although, the efficiency of Di-Fe/Ni for removal of OII is higher in lower pH ranges, it shows significant removal efficiency (>93.00%) at higher pH ranges, even at pH 11. This indicates that Di-Fe/Ni nanocomposite has high capability for OII removal at a wide pH range. This discovery is one advantage of this process over Fenton reaction, which requires low working pH (range of 2–3) to attain maximum removal efficiency.^15^ On the other hand, for Fe/Ni, OII removal efficiency increases from 74.83 to 90.81% when the pH decreases from 11 to 3, indicating that acidic condition is favourable for OII removal. When decreasing initial pH of solution (increasing acidity), the corrosion of Fe induces the formation of H^+^ and then H_2_ at Ni surface.^14^ This latter process could be a key factor for the removal of OII through adsorption by the nanoparticles followed by hybrid transmission from the surface of Ni.^14^ The increase in the removal efficiency under acidic conditions may be elucidated based on reactions happening on the surface of Fe/Ni (eqn (4)–(8)):^14^

4 Fe–Ni + H^+^ ⇋ Fe^+^–Ni + H˙

5 Fe^+^–Ni + H^+^ ⇋ Fe^2+^–Ni + H˙

6 Fe^2+^–Ni ⇋ Fe–Ni^2+^

7 H˙ + H˙ ⇋ H_2_

8 2Fe–Ni + H_2_ ⇋ 2Fe–Ni–H

However, the reactivity of Fe/Ni is lower at higher pH values (alkaline condition) owing to the formation of hydroxide layers on the surface of these nanoparticles.^14^ Our results on the effect of pH are in accord with previous literature.^14,18^

#### 3.3.2 Effect of catalyst amount on OII degradation

**Fig. 7:**
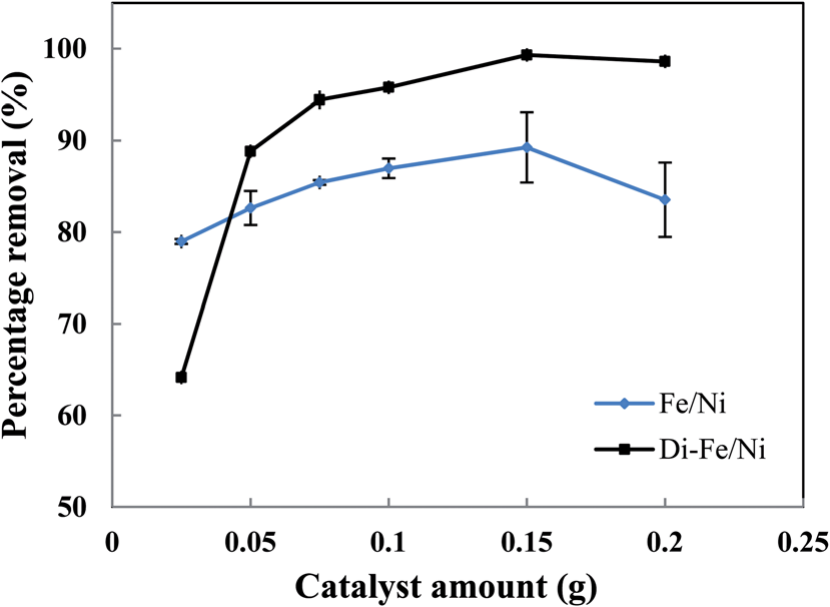
Effect of catalyst amount on OII removal using Fe/Ni and Di-Fe/Ni (initial concentration of OII: 100 mg L^−1^; volume of OII solution: 25 mL; pH: 6.84; reaction time: 6 h).

The effect of catalyst amount on OII removal efficiency is presented in Fig. 7. Generally, Di-Fe/Ni has superior efficiency than Fe/Ni with regards to removal of OII. The maximum removal percentages of Di-Fe/Ni and Fe/Ni (in 25 mL of OII solution, initial OII concentration of 100 mg L^−1^, and with 0.150 g of catalyst amount) are 99.31 and 89.26%, respectively. This outcome is consistent with the deduction that Di as a support material decreases the aggregation of Fe/Ni nanoparticles, thereby increasing their reactivity toward OII.

The OII removal efficiency increases with use of greater catalyst amount from 0.025 to 0.150 g, but it decreases significantly when the quantity increases further to 0.200 g (especially for Fe/Ni). This behaviour is correlated with a distinct increase in the pH of solution (as seen in Table S1†), which hinders the oxidation reaction. In addition, there is a noted increase in removal efficiency by Di-Fe/Ni from 64.15 to 88.83% when the amount increases from 0.025 to 0.050 g, indicating that the sample amount lower than 0.050 g is not favourable for the removal of OII by the composite.

In conclusion, the optimum amount for both Di-Fe/Ni and Fe/Ni was determined as 0.150 g in 25 mL of solution (6 g L^−1^). For Di-Fe/Ni, the increase in catalyst amount can result in an increase in removal efficiency, as this material does not significantly influence the solution pH. In contrast, Fe/Ni increases the pH of solution with final value depending on catalyst amount.

#### 3.3.3 Effect of initial dye concentration on OII degradation

**Fig. 8:**
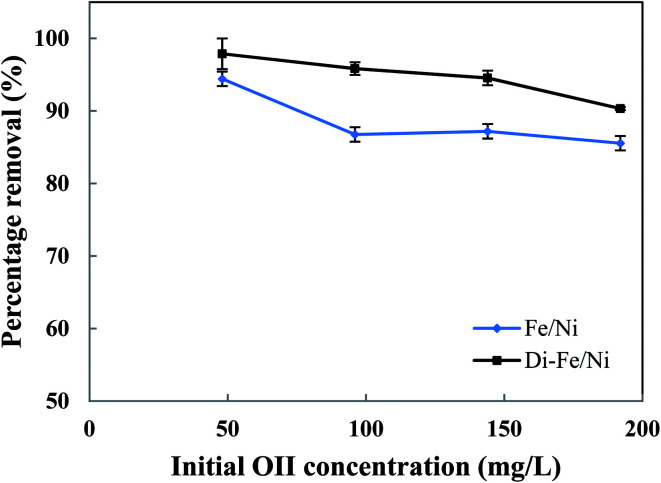
Effect of initial dye concentration on OII removal using Fe/Ni and Di-Fe/Ni (volume of OII solution: 25 mL; catalyst amount: 0.1 g; pH: 6.84; reaction time: 6 h).

Initial OII concentration is another factor, which, in theory, could affect the removal efficiency. The influence of initial OII concentration on removal efficiency by Di-Fe/Ni and Fe/Ni was investigated with 0.1 g of catalyst amount, 25 mL of OII solutions and at natural pH. The initial concentration of OII was varied between 50 to 200 mg L^−1^. As observed from Fig. 8, for both samples, the highest removal percentage was achieved using 50 mg L^−1^ of initial OII concentration, as 97.88 and 94.42% of OII were removed by Di-Fe/Ni and Fe/Ni, respectively.

Moreover, with a decrease in the initial OII concentration the removal efficiency increases, within 6 h of reaction. For instance, the removal efficiency by Di-Fe/Ni increases from 90.33 to 97.88% when the initial OII concentration decreases from 200 to 50 mg L^−1^. Generally, the removal of OII by Di-Fe/Ni is a heterogeneous reaction, which includes adsorption of OII on the surface of Ni followed by surface reaction.^14^ Increasing the initial OII concentration would result in competitive adsorption between the OII molecules when the number of adsorption sites of Di-Fe/Ni is limited. Similar results were observed in a study, which used bentonite-supported Fe/Ni composite for the removal of amoxicillin.^18^

#### 3.3.4 Effect of reaction time on OII degradation

**Fig. 9:**
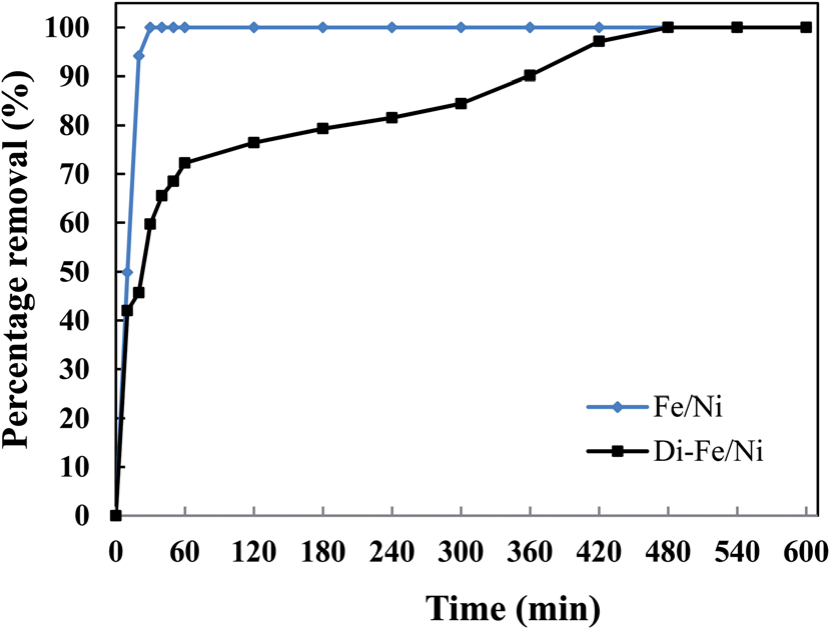
Effect of reaction time on OII removal using Fe/Ni and Di-Fe/Ni (initial concentration of OII: 100 mg L^−1^; volume of OII solution: 250 mL; catalyst amount: 1.0 g; pH: 6.84).

The influence of reaction time on removal of OII by Di-Fe/Ni and Fe/Ni was performed using 1 g of each catalyst with 250 mL of 100 mg L^−1^ OII solution at natural pH. The mixtures were stirred at room temperature for certain periods of time (10, 20, 30, 40, 50, 60, 120, 180, 240 and up to 600 min) (Fig. 9). After the reaction, 5 mL of the supernatant was withdrawn using a syringe with a hydrophilic PTFE filter (0.22 μm). Dye concentration was measured spectrophotometrically using an Agilent Cary 60 UV-Vis Spectrophotometer by measuring absorbance at *k*_max_ of 485 nm. The amount of OII removed was calculated using eqn (3). Firstly, for both catalysts, increasing the reaction time promotes the OII removal efficiency. Moreover, the equilibrium concentrations were found to be at the same level, showing that the removal efficiencies for the two catalysts are comparable. The OII concentration decreases from 97.9 mg L^−1^ to lower than 1.0 mg L^−1^.

The complete removal of OII was reached faster by Fe/Ni, while for Di-Fe/Ni it requires a longer reaction time. For Fe/Ni, there is a sharp drop in the concentration of OII during the first 20 min of reaction, as only 5.76 mg L^−1^ of OII remains in solution, indicating 94.12% removal efficiency. However, for Di-Fe/Ni nanocomposite, the OII concentration decreases gradually with 2.81 mg L^−1^ of OII remaining in the solution after 420 min of reaction (97.13% removal efficiency). Although, Di-Fe/Ni contains a significantly lower amount of the bimetallic nanoparticles (≤16 wt%), it could completely remove OII from the solution. The reaction rate of Di-Fe/Ni can be enhanced by loading more bimetallic particles but it brings along more disadvantages. For example, dispersion a high volume of Fe/Ni is harmful to the environment. The results attained form Fig. 5 indicate that Di alone does not exhibit significant OII removal, as only 3.59% of OII was removed through presumably adsorption. This low adsorption capability may be due to the presence of negative surface charge of Di which repels negative sulfonate groups of OII. However, the removal of OII was significantly increased (100% removal efficiency) when Di was modified by Fe/Ni nanoparticles on the surface of Di-Fe/Ni. Loading of Fe/Ni on the surface of Di significantly improves their catalytic reactivity due to increased dispersion of the particles.

### 3.4 Removal of total organic carbon

**Fig. 10:**
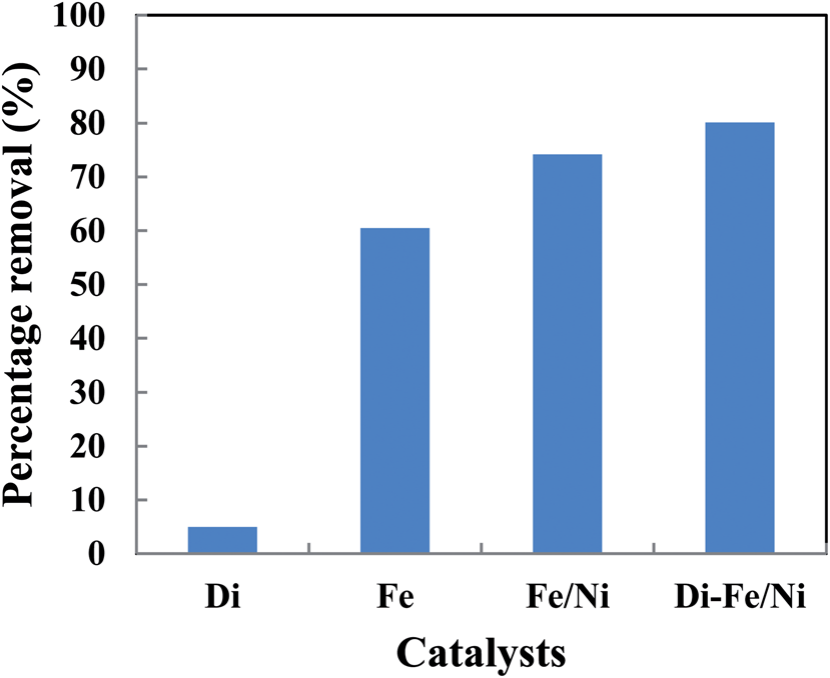
The removal of TOC using different catalysts (initial concentration of OII: 100 mg L^−1^; volume of OII solution: 25 mL; catalyst amount: 0.1 g; pH: 6.84; reaction time: 6 h).

The removal of total organic carbon (TOC) using different catalysts is displayed in Fig. 10. The results show that the removals of TOC by Di-Fe/Ni, Fe/Ni and Fe are 80.12, 74.17 and 60.44%, respectively. Moreover, Di alone has minimal impact on TOC removal due to its low adsorption capacity, as only 4.94% of the TOC was removed. The removal of TOC significantly increases when Di was modified by Fe/Ni nanoparticles. Hence, TOC can be effectively removed through a synergistic adsorption–degradation process on the surface of Di-Fe/Ni. It is suggested that the combination of hydrogen radicals (H˙) with OII molecules can break down the chromophore group and conjugated system of OII. Consequently, at the end of the experiment, low proportion of the initial TOC remains in solution. This result indicates that the majority of OII has been reduced and removed through catalytic degradation process rather than adsorption by using Di-Fe/Ni nanocomposite (the stable carbon mass percentage of the composite material before and after reaction as discussed later in this paper supports this conclusion).

### 3.5 Removal mechanism

**Fig. 11:**
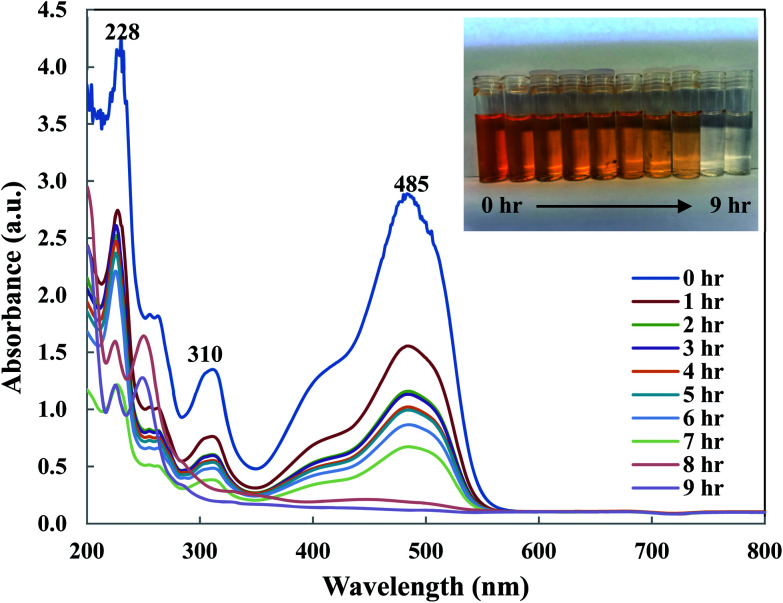
UV-Vis spectrum of OII solution recorded at different reaction times.

To understand OII removal mechanism by Di-Fe/Ni nanocomposite, UV-Vis and micro organic analyses (carbon mass percentages of Di-Fe/Ni) were used in this study. Fig. 11 shows the UV-Vis spectrum of OII recorded at different reaction times. There are three characteristic wavelengths for OII at 228, 310 and 485 nm, corresponded to benzene ring, naphthalene ring and azo bond, respectively. As shown in Fig. 11, absorbance of these three wavelengths becomes lower, as there are sharp drop for the azo bond and slight shifts for the benzene and naphthalene ring. The sharp drop of absorbance at 485 nm indicates that the azo bond was destroyed or adsorbed onto surface of Di-Fe/Ni. Moreover, the colour of solution changes from orange to colourless. As azo bond is the basic reason for the visible colour of OII, this colour changes can confirm the cleavage of the azo bond. These outcomes do not indicate that the removal mechanism involves only adsorption, as the adsorption of degraded products onto the surface of Fe/Ni has been reported.^32^

In order to determine the dominant process for the removal of OII, carbon mass percentages (C mass%) of Di-Fe/Ni nanocomposite were measured before and after reaction using micro organic analyser. C mass% of Di is 0.24%; while, after loading Fe/Ni nanoparticles, it slightly increases to 0.25% albeit this value is within error measurements of the system. There is also a negligible increase in C mass% (less than 0.01%) for Di-Fe/Ni after reaction with OII, indicating that only a small amount of carbon may be adsorbed onto the surface of Di-Fe/Ni. Hence, the catalytic degradation is probably dominant when removing OII by Di-Fe/Ni nanocomposite. This conclusion has also been confirmed by TOC analysis, which shows that the carbon content has been significantly degraded after reaction, indicating most OII was removed through catalytic reduction rather than adsorption.

There are two main removal mechanisms of OII, namely adsorption by Di/Fe–Ni nanocomposite followed by catalytic reduction by Fe/Ni. Particularly, the first pathway describes that OII was adsorbed onto the surface of composite material. Cationic OII molecules may be adsorbed onto the catalyst surface due to physico-chemical properties such as high porosity. The next and the most significant pathway is a reduction process on Fe/Ni bimetallic system, where nZVI (Fe^0^) generates hydrogen through a corrosion reaction and Ni acts as a hydrogenation catalyst, which firstly enhances the corrosion rate of Fe^0^ and then adsorbs H_2_. Subsequently, H_2_ can be dissociated on the Ni surface, resulting in the formation of nickel hydride (Ni–H) and hydrogen radicals (H˙). The hydrogen radicals were then combined with OII molecules and induced the cleavage of the azo bond, finally broke the conjugated structure and chromophore group of OII. Therefore, the role of Ni loading on the Fe^0^ surface is to provide active sites in the bimetallic system. The heterogeneous removal of OII by Di-Fe/Ni nanocomposite can be described as follows:

(1) Adsorption, production of H_2_ and corrosion of iron (eqn (9)–(11)):

9 OII + diatomite → OII–diatomite (adsorption)

10 Fe^0^ + 2H^+^ → Fe^2+^ + H_2_ (in acidic conditions)

11 Fe^0^ + 2H_2_O → Fe^2+^ + H_2_ + 2OH^−^ (in alkaline conditions)

(2) The dissociation of H_2_ on Ni surface, resulting in the formation of Ni–H or H˙ for the reduction reaction (eqn (12) and (13)).

12 2Ni^0^ + H_2_ → 2Ni–H

13 Ni–H → Ni^0^ + H˙

(3) Combination of H˙ with OII molecules and breaking down the chromophore group and conjugated system of OII, and finally the reduction of OII into 1-amino-2-naphthol and sulphanilic acid.^32^

**Fig. 12:**
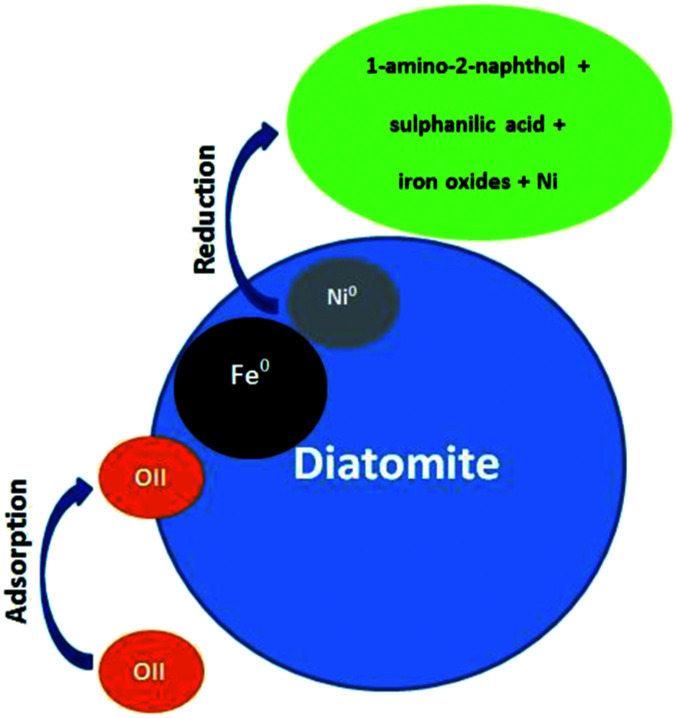
Conceptual model of the OII removal mechanism using Di-Fe/Ni.

The OII removal mechanism using Di-Fe/Ni based on the above-mentioned results and discussion is summarised in Fig. 12.

## 4 Conclusions

A nanocomposite material, Di-Fe/Ni, was successfully synthesised through a reduction method. Di-Fe/Ni can effectively degrade OII in aqueous solution by the cleavage of azo bond, and breaking down of its chromophore group and conjugated system. XRD, XPS, SEM and TEM analysis confirmed that bimetallic Fe/Ni nanoparticles loaded onto diatomite are well dispersed thereby enhancing the reactivity. Batch degradation experiments indicate that initial pH of the solution, catalyst amount, initial OII concentration and reaction time are the main parameters, significantly affecting OII removal efficiency. Optimum operational parameters determined are pH 3, catalyst amount of 0.150 g and initial OII concentration of 50 mg L^−1^. A synergistic process was proposed for the degradation of OII, including both adsorption and catalytic reduction. Finally, 99.00% OII removal efficiency and 80.12% TOC removal demonstrate the promise of Di-Fe/Ni nanocomposite in the remediation of organic contaminant polluted wastewater.

## Conflicts of interest

There are no conflicts to declare.

## Abbreviations

XRD: X-ray diffraction
XPS: X-ray photoelectron spectroscopy
TEM: Transmission electron microscope
SEM: Scanning electron microscopy
OII: Orange II
AOPs: Advanced oxidation processes
nZVI: Nanoscale zero-valent iron
Di-Fe/Ni: Diatomite-supported Fe/Ni
TOC: Total organic carbon
LA-ICP-MS: Laser ablation inductively coupled plasma mass spectrometry
Di: Diatomite

## Supplementary Material

RA-008-C7RA13348K-s001
