# Supplementary material for: Catalytic degradation of Orange II in aqueous solution using diatomite-supported bimetallic Fe/Ni nanoparticles
Source: RSC Adv. 2018 Feb 16;8(14):7687–96. doi: 10.1039/c7ra13348k (PMC9078455; doi:10.1039/c7ra13348k)
Supplement: RA-008-C7RA13348K-s001 [file RA-008-C7RA13348K-s001.pdf]

Electronic Supplementary Material (ESI) for RSC Advances.
This journal is © The Royal Society of Chemistry 2018

Supplementary

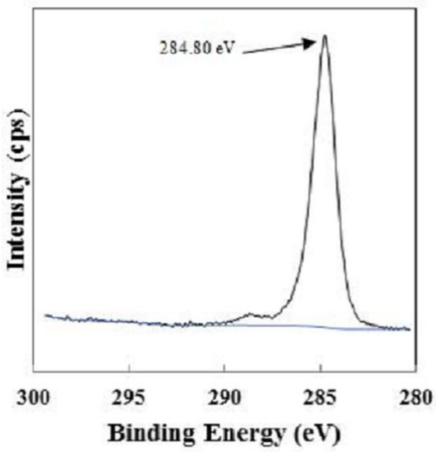

Figure S1 XPS spectra (C 1s) of Di-Fe/Ni composite.

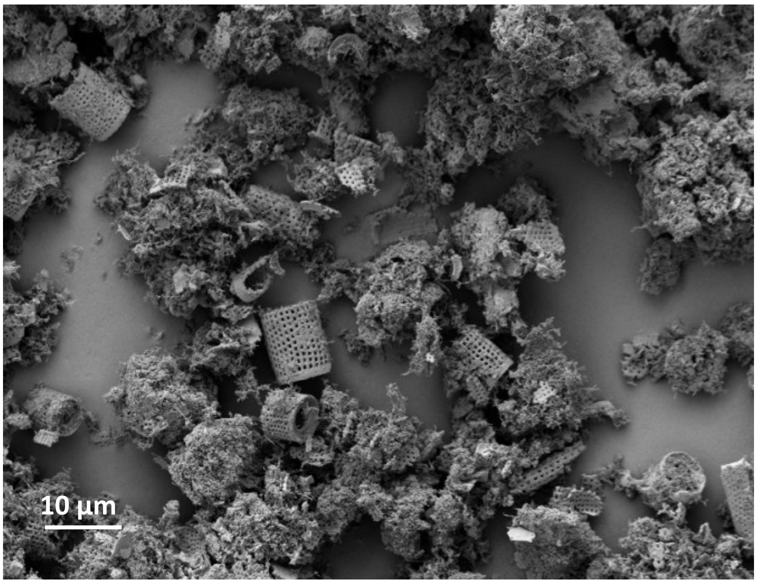

Figure S2 SEM image of Di-Fe/Ni.

Table S1 pH after reaction for Fe/Ni and Di-Fe/Ni.

	**pH after reaction**	
**Catalyst amount (g)**	**Fe/Ni**	**Di-Fe/Ni**
0.025	9.34	8.21
0.050	9.38	8.25
0.075	9.41	8.26
0.100	9.42	8.33
0.150	9.48	8.39
0.200	9.89	8.70
